# Circadian Biology in Obstructive Sleep Apnea

**DOI:** 10.3390/diagnostics11061082

**Published:** 2021-06-13

**Authors:** Bala S. C. Koritala, Zachary Conroy, David F. Smith

**Affiliations:** 1 Division of Pediatric Otolaryngology-Head and Neck Surgery, Cincinnati Children’s Hospital Medical Center, Cincinnati, OH 45229, USA; Bala.Koritala@cchmc.org; 2 College of Medicine, University of Cincinnati, Cincinnati, OH 45267, USA; Conroyze@mail.uc.edu; 3 Department of Otolaryngology-Head and Neck Surgery, University of Cincinnati College of Medicine, Cincinnati, OH 45267, USA; 4 Division of Pulmonary Medicine, Cincinnati Children’s Hospital Medical Center, Cincinnati, OH 45229, USA; 5 The Sleep Center, Cincinnati Children’s Hospital Medical Center, Cincinnati, OH 45229, USA; 6 The Center for Circadian Medicine, Cincinnati Children’s Hospital Medical Center, Cincinnati, OH 45229, USA

**Keywords:** circadian rhythms, OSA, biomarkers, genes, metabolites

## Abstract

Obstructive sleep apnea (OSA) is a complex process that can lead to the dysregulation of the molecular clock, as well as 24 h rhythms of sleep and wake, blood pressure, and other associated biological processes. Previous work has demonstrated crosstalk between the circadian clock and hypoxia-responsive pathways. However, even in the absence of OSA, disrupted clocks can exacerbate OSA-associated outcomes (e.g., cardiovascular or cognitive outcomes). As we expand our understanding of circadian biology in the setting of OSA, this information could play a significant role in the diagnosis and treatment of OSA. Here, we summarize the pre-existing knowledge of circadian biology in patients with OSA and examine the utility of circadian biomarkers as alternative clinical tools.

## 1. Introduction

The circadian system has been an integral part of evolution for predicting environmental changes and maintaining organisms’ health and fitness [1]. Almost all cells and organs in the human body express molecular, biochemical, and physiological circadian patterns [2]. These patterns respond to light exposure, daily activity, the timing of food intake, and other health conditions [3]. In the real world, disrupted rhythms are observed in humans who receive abnormal light exposure and have irregular sleep and eating habits. Studies in humans and rodents suggest that circadian disruption is associated with significant health conditions [4,5,6,7]. However, the relationship between circadian disruption and human health remains a “chicken or the egg” question.

Sleep is a predominant circadian phenotype in humans. Disrupted sleep potentially dysregulates circadian rhythms, impacts multiple biological and physiological processes, and likely contributes to disease initiation or progression [8]. In this review, we focus on circadian biology in obstructive sleep apnea (OSA). OSA is highly prevalent among children and adults and is characterized by upper airway obstruction and intermittent hypoxia during sleep. Patients with OSA typically experience cyclical patterns of oxygen desaturation followed by reoxygenation during their sleep phase [9,10]. The pathophysiological consequences of OSA are associated with the rhythmic dysregulation of sleep patterns and blood pressure (BP). Additionally, sleep fragmentation can be a potential hallmark for this health condition [11]. Recent studies have shown that exposure to hypoxic conditions can alter circadian gene expression and associated biological processes [12,13,14,15]. More importantly, chronic exposure to hypoxic conditions can lead to disorders that are commonly associated with the dysregulation of circadian rhythms including cardiovascular diseases, respiratory diseases, dementia, cancer, and metabolic disorders [16,17,18].

Since ~50% of genes are rhythmic in mammals [19,20,21], the circadian clock has implications for disease regulation, but also in response to therapeutic intervention and prognosis [22,23,24,25]. Though several studies have demonstrated time-specific treatment efficacy, the majority of these studies are limited to cancer therapeutics [26]. These observations suggest that circadian medicine is still underdeveloped, and further research is needed to understand its role in a wide range of health conditions. In this context, we recognize the importance of circadian biology in the processes associated with OSA. The aim of this literature review is to summarize the pre-existing knowledge on circadian rhythms and OSA and to provide guidance on potential areas of research in clinical diagnostics and therapeutics for patients with OSA.

## 2. Circadian Rhythms in Humans

In humans, circadian rhythms are maintained through a natural cycle of 24 h light and dark. Intrinsically photosensitive retinal ganglion cells (IPRGCs) in the human eye receive light and pass this information to the central oscillator (suprachiasmatic nuclei) located in the hypothalamus [27,28]. This information serves to entrain, or synchronize, the circadian system to the environment. In addition, peripheral, cell-autonomous clocks are located throughout the body [29]. These peripheral clocks coordinate with the central oscillator and maintain circadian homeostasis of the human body by regulating multiple physiological outputs including the sleep–wake cycle, core body temperature, BP, heart rate, hormone secretion, etc. [30,31].

At the cellular level, the circadian clock functions as a transcriptional and translational feedback loop by self-regulating its own activity [32,33]. The key transcription factor, BMAL1, interacts with CLOCK and its paralog NPAS2, along with other clock transcription factors, to activate circadian gene expression of hundreds of their target genes including their own repressors PER1, PER2, PER3, CRY1, and CRY2. The transcriptional activation and repression of the clock is controlled through E-BOX elements, locations where transcription factors promote gene expression. It takes approximately 24 h to complete this feedback loop. In addition to PERs and CRYs, RORs and REV–ERBs activate and repress transcriptional activation of BMAL1, respectively. Furthermore, DEC1, DEC2, and kinases provide stability to this complex oscillatory network. These “canonical clock” genes influence the expression of thousands of other genes, proteins, and associated key biological pathways including cell cycle, DNA repair, immune responses, and metabolism [34,35,36,37].

Changes in these molecular and physiological rhythms often serve as diagnostic tools for disease.

## 3. Physiological Rhythms in OSA

For decades, sleep–wake patterns, BP, melatonin, cortisol, and core body temperature have been extensively used as biomarkers for measuring circadian physiology in humans [38,39,40,41,42]. In fact, most studies evaluating circadian biology in OSA have used these standard circadian markers.

As mentioned, fragmented sleep is a potential hallmark of OSA that occurs due to repeated episodes of arousals [11]. Disrupted sleep patterns not only influence the quality and quantity of sleep but also increase the severity of OSA [43]. Polysomnography (PSG) is the gold standard test for the diagnosis of OSA and is used to measure the apnea–hypopnea index (AHI) [44]. However, PSG is also used to measure changes in sleep patterns among patients with OSA by evaluating other parameters such as total sleep time, sleep efficiency, and the arousal index. Accumulated evidence suggests that many disease states are worsened by a lack of sleep and/or circadian misalignment. Likewise, other health consequences, such as cardiovascular dysfunction and cognitive changes, that occur in the setting of disrupted sleep or circadian rhythms have also been reported in untreated OSA [45,46,47,48,49,50]. However, these physiological abnormalities may also be influenced or worsened by hypoxemia during sleep. Interestingly, the OSA-associated risk to the cardiovascular and central nervous systems are very similar to those resulting directly from disrupted sleep in the absence of hypoxic events [51]. Although the underlying mechanism is unknown, several studies have reported irregular BP rhythms in patients with OSA [52]. Systolic and diastolic BP exhibit 24 h rhythms in healthy humans with peaks of BP occurring in the morning and afternoon and a notable trough occurring during sleep [38]. Changes in these 24 h rhythmic patterns have been reported in patients with OSA, and this could be associated with the cardiovascular abnormalities known to occur in this patient population [53,54,55,56,57,58,59,60,61].

Cortisol is a hormone that exhibits circadian rhythm and is involved in BP regulation, immune responses, and metabolism. Sleep disruption influences hypothalamic–pituitary–adrenal (HPA) activity and increases cortisol secretion [42]. Although several studies have evaluated cortisol secretion in patients with OSA [62,63,64,65,66,67,68,69,70], these studies were unable to consistently demonstrate significant changes in the setting of OSA due to inconsistent observations and/or methodological constraints including sampling time, sampling frequency, and analytic techniques. Melatonin is another hormone that exhibits circadian rhythmicity, with an increase occurring during sleep. Melatonin is involved in sleep regulation and is frequently used to promote sleep onset in patients with insomnia [41,71]. Melatonin measurements (DLMO, dim light melatonin onset) have been widely used to understand circadian phases in human sleep and in circadian rhythm sleep-wake disorders [72]. Therefore, changes in melatonin levels have also been evaluated to better understand changes in circadian biology in patients with OSA. Although, melatonin secretion is rhythmic in OSA patients [73] and may vary among patients with and without OSA [74], a significant number of studies have shown no changes in the rhythmic patterns of melatonin secretion/excretion or in response to dim-light onset among those with OSA compared to healthy controls [69,75,76,77]. These studies suggest that hormones such as melatonin and cortisol are not preferable as circadian markers for the diagnosis and treatment of OSA.

Only a limited number of studies have reported on body temperature in patients with OSA [68,78,79,80]. No variations have been seen in body temperature rhythms between patients with OSA and healthy controls [80]. However, treatment of OSA with continuous positive airway pressure (CPAP) did not lead to phase differences in body temperature, but did impact the amplitude [68,78,79].

## 4. Molecular Rhythms in OSA

There is an overlap in canonical clock genes and associated biological processes, such as inflammation, metabolism, cell cycle, apoptosis, DNA repair, redox signaling, and oxidative stress, in disease processes of multiple health conditions including cancer, dementia, metabolic, cardiovascular, and hypoxemic disorders [81,82,83]. OSA is a hypoxemic disorder associated with increased oxidative stress and the upregulation of systemic inflammatory responses [84]. Pro-inflammatory cytokines, TNFα and IL-6, have been extensively investigated in patients with OSA [85]. TNFα and IL-6 both exhibit circadian rhythmicity [86]. TNFα in particular demonstrates altered circadian rhythms in patients with OSA [75]. Furthermore, diurnal variation of plasma cytokines (TNFα, IL-6, and IL-8) were identified in children with severe OSA compared to healthy controls [87]. Upregulation of these systemic inflammatory markers is strongly associated with morbid phenotypic signatures in patients with OSA.

A primary regulator of oxygen homeostasis in mammals is hypoxia inducible factor-1 (HIF-1). Under hypoxic conditions, HIF-1α heterodimerizes with HIF-β and binds to the E-box of hypoxia response elements to promote the expression of multiple target genes associated with cellular and biochemical processes [88]. A few studies have shown bidirectional interactions of HIF-1 with the circadian clock [89,90]. For example, BMAL1 and CLOCK heterodimerize and regulate the rhythmic expression of *HIF-1α* [90]. Interestingly, HIF-1α colocalizes with BMAL1 and regulates the expression of *CRY1* and PER2 [89]. As a feedback regulator, CRY1 and PER2 interact with HIF-1α and alter hypoxia-induced responses. PER2 promotes the activity of HIF-1 [91], and CRY1 suppresses transcriptional activity of HIF-1α [92,93]. One possible reason for bidirectional interactions is the presence of E-box sites on *HIF-1α*, *PER*, and *CRY* genes [32,90]. A number of studies have reported an increase of HIF-1α in patients with OSA compared to healthy individuals [94,95,96,97,98]. A recent study has shown increased levels of HIF-1α in patients with OSA, correlating with an increased expression of clock proteins [98]. Taken together, prime circadian oscillatory mechanisms are intertwined with hypoxia inducible factors. These interactions could potentially alter the function of the circadian system under hypoxic conditions [15]. This hypothesis was supported by recent -omics studies in rodents. These studies demonstrated the alteration of the circadian system by time and tissue-specific hypoxia responses under short term exposure to intermittent hypoxia [13,14].

Several studies have tried to specifically evaluate the responses of canonical clock genes in patients with OSA [68,99,100,101,102,103]. Burioka et al. have demonstrated the arrhythmic expression of *PER1* in patients with OSA. However, treatment with CPAP recovered PER1 rhythms similar to those in healthy controls [100]. A time course study with a 24 h gene expression profile of nine clock genes showed arrhythmic *BMAL1*, *CLOCK*, and *CRY2* in patients with OSA. In this study, the authors correlated gene expression profiles with the AHI index, and their observations suggest that the reduction of *CRY1* and *PER3* levels at specific time points (e.g., mid-night) may serve as predictors for those with severe OSA [99]. Another time course study with eleven clock genes has shown the alteration of time-specific expression of *BMAL1*, *PER1*, *CRY2*, and *DEC1* in patients with OSA. However, these altered expression patterns were not recovered by short or long-term CPAP treatments [68]. Additionally, a few studies have reported clock gene expression in blood from single time points for patients with OSA. Moreira et al. have investigated the expression of seven canonical clock genes and identified a decrease in *CLOCK* gene levels in patients with OSA. Furthermore, no recovery was observed after treatment with CPAP [101]. Additional work by Canales et al. showed clock gene dysregulation in both a sleep apnea and a nocturnal hypoxemia cohort compared to their controls [103]. Interestingly, those patients with nocturnal hypoxemia had more dysregulated clock genes than those patients with OSA.

## 5. Circadian Rhythms in Diagnostics and Therapeutics of OSA

The diagnosis of OSA can be quite challenging and is often entirely dependent on the availability of sleep labs and portable sleep studies. There are no physical assessments that can be used to determine the severity of OSA and an overnight PSG is the only standard tool available for diagnosis. Although OSA has been recognized as a highly prevalent health condition, the field of diagnostics is limited and clinical focus is poorly developed [43,44]. Undiagnosed or untreated OSA in children and adults may impact multiple organ systems and has a high morbidity and mortality burden [9,104]. PSG is not an economical process and can be quite time-consuming for patients and healthcare systems alike. This is especially true for those healthcare systems in underdeveloped countries. Furthermore, a single-night PSG has a relatively weak test–retest reliability, an important consideration for the reliability of the diagnosis of OSA [105]. Therefore, a simple, cost-efficient, robust, and rapid test would be a significant advancement for the diagnosis and treatment of OSA. In modern diagnostics and treatment regimens, gene expression and metabolites play a significant role for several health conditions [106,107,108]. However, gene expression varies with time of day, as ~50% of the mammalian transcriptome exhibit circadian rhythms [19,20,21]. In addition, metabolites also exhibit rhythmicity in humans [109,110]. Therefore, we think gene expression and metabolite rhythmicity may play a significant role in the development of biomarkers, the timing of diagnosis, and the timing of treatment for multiple health conditions, including OSA.

Rodent studies have demonstrated that physiological oxygen fluctuations may alter circadian rhythms and the time-dependent expression of RNA and proteins in cells and tissues [13,14,110]. Likewise, studies of clock genes in humans have demonstrated the alteration of both rhythmic and time-dependent gene expression profiles in patients with OSA [68,99,100]. Although there is still a gap to understand the mechanisms of circadian biology and OSA in humans, rodent studies suggest that oxygen fluctuations alter the expression of rhythmic clock genes through HIF-1α [111]. Furthermore, a number of studies also showed recovered molecular and/or physiological rhythms after CPAP therapy in patients with OSA [68,79,99]. These observations suggest that circadian patterns could serve as potential biomarkers for the diagnosis and treatment of OSA.

## 6. Future Directions

**Figure 1:**
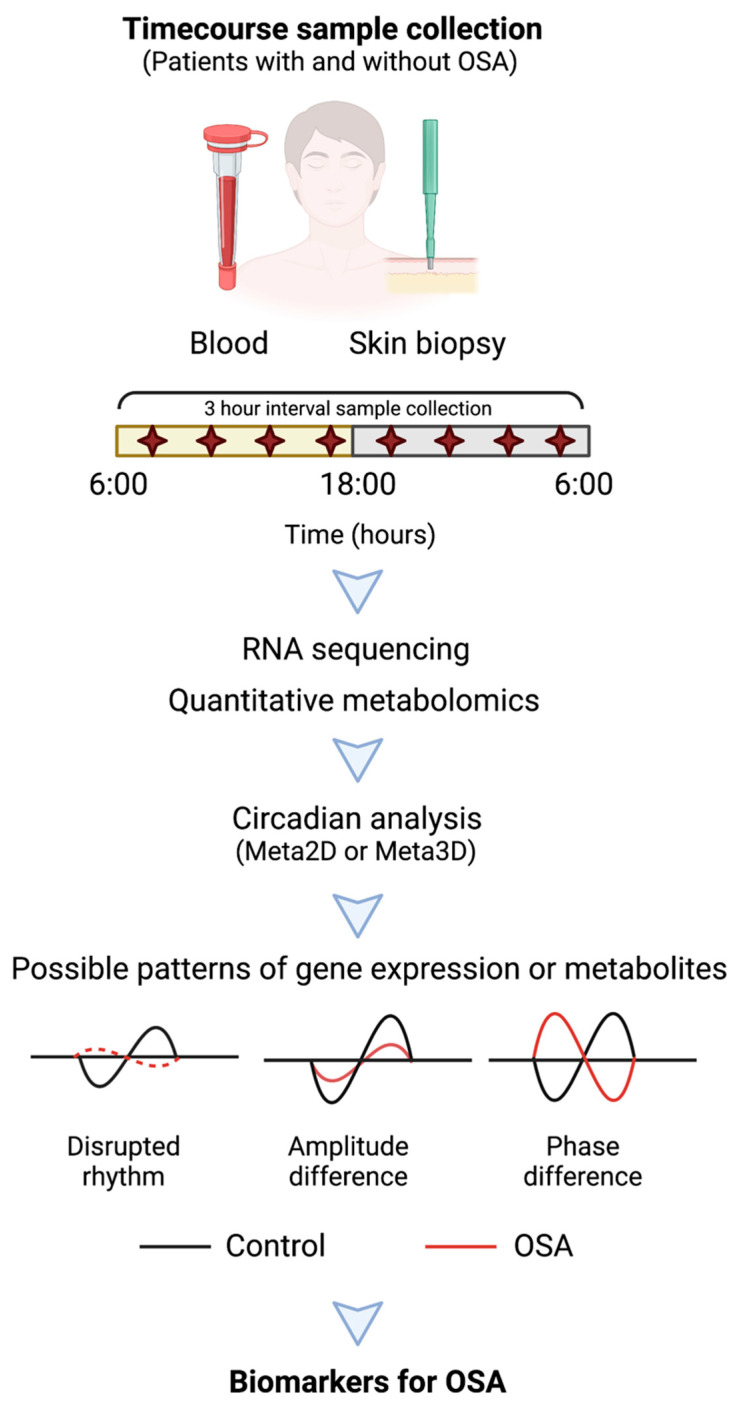
A study design for developing circadian biomarkers in patients with OSA. Blood or skin samples should be collected over a period of 24 h from patients with and without OSA. Extracted RNA or metabolites further processed for RNA sequencing or quantitative metabolomics. Analytical tools such as MetaCycle (Meta2D and 3D) should be used to detect rhythms of transcriptome or metabolites among patients with and without OSA. Genes or metabolites with amplitude or phase differences among healthy controls vs. patients with OSA may be used as potential biomarkers for OSA diagnosis and treatment. This figure was created with BioRender.com.

The development of biomarkers requires a system-level understanding of gene expression and its role in disease processes. So far, there are no time course studies available that specifically evaluate the circadian transcriptome, circadian proteome, or circadian metabolome in patients with OSA. The available circadian studies are mainly targeted to understanding the expression of canonical clock genes. Therefore, we believe it is worthwhile to further evaluate circadian transcriptome or metabolome expression profiles in patients with OSA and to develop time-dependent biomarkers for use in OSA-associated diagnostics and therapeutics. Here, we provide a strategy to develop circadian biomarkers for these purposes (Figure 1).

There are two possible and less invasive methods for developing robust circadian biomarkers in humans, blood collection and skin biopsy. A recent study has shown that the skin clock is robust compared to the circadian clock in blood [112]. However, the most appropriate tissue for use in circadian biomarker studies is unknown. Studies have shown mechanistic links between OSA and diseases of the skin [113,114]. In parallel, studies have shown altered circadian rhythms of canonical genes in the blood of patients with OSA. In fact, monocytes are a source of robust circadian biomarkers [115]. Additionally, metabolite biomarkers can be developed using plasma [109]. Taken together, both skin and blood have the potential to serve as a source of OSA-associated biomarkers.

To develop circadian or time-dependent biomarkers for OSA, we recommend conducting time course studies with at least 3 or 4 h interval sample collections of blood or skin for a period of 24 h from patients with and without OSA. For a comprehensive study design, guidelines for the use of circadian -omics data have already been outlined by experts in the field of circadian biology [116]. Samples from both healthy controls and subjects with OSA should be processed for circadian analysis of both the transcriptome and metabolites. There are multiple ways to measure circadian rhythms with both advantages and disadvantages to these methods [117]. However, this study design would help to understand the alteration of the circadian transcriptome or metabolome among subjects with and without OSA for direct comparison. A systemic alteration of rhythmic genes or metabolites in patients with OSA may serve to identify targets for further diagnostic value (Figure 1).

How would we then use these biomarkers in real-world clinical applications? Once reliable biomarkers are established, we suggest measuring candidate biomarkers from at least two different times of the day based on the peak and trough patterns of their rhythmic expression. This strategy may lead to the identification of circadian biomarkers with significant clinical use for patients with OSA.
